# Assessment of consumer perspectives on the use of paper packaging in Trinidad

**DOI:** 10.1371/journal.pone.0323456

**Published:** 2025-05-13

**Authors:** Farrah Mathura, Rohanie Maharaj

**Affiliations:** Department of Chemical Engineering, University of the West Indies, St. Augustine, Trinidad; University of Georgia, UNITED STATES OF AMERICA

## Abstract

**Background:**

Paper packaging is increasingly being used to replace single use plastics to mitigate the negative effects of plastic pollution on the environment. While many developed countries have made considerable strides in this transition, developing countries, like Trinidad, have lagged behind, partly, due to no stringent legislation for types of packaging at commercial outlets.

**Objectives:**

The objectives of this study were to investigate the demand for and assess public perceptions of sustainable paper packaging in Trinidad.

**Methodology:**

Data were collected using a Knowledge, Attitudes, Practices (KAP) survey supplemented by interviews, packaging stress testing, and analysis via SPSS.

**Results:**

A majority of participants demonstrated awareness of recycling knowledge (79%) and deforestation awareness (84%); which aligns with the 71% of participants who considered themselves environmentally conscious. The attitudes and practices of participants did not correlate directly with their environmentally conscious behaviour such as willingness to purchase paper-based food containers (67%), finding paper packaging more aesthetically pleasing than plastic (73%) and willingness to pay more for paper packaging (53%). The interviews highlighted a demand for sustainable paper packaging while also identifying challenges such as waterlogging of packaging, sustainable raw materials sourcing, packaging strength, and the need for customer testing and feedback. The stress test showed good appearance and durability.

**Conclusions:**

Overall, the sustainable paper packaging stress test received favourable feedback and there were varying levels of knowledge, attitudes and practices related to sustainable packaging. Future research can build on the current findings and the questionnaire can be replicated after the introduction of replacement of single use plastics with non-wood paper packaging.

## Introduction

Packaging is the greatest contributor to waste worldwide with about 97% being unrecycled [1]. Plastic pollution from single use plastics (SUPs) is a growing problem. For SUPs such as straws, boxes, containers and plates, the lifespan can be as short as a few minutes. The high velocity of use of SUPs is problematic as there is a corresponding large accumulation of plastic waste in the environment. The large amount of waste has become unmanageable in many developing countries as a significant portion of the volume is air which depletes space quickly and upon degradation, there is are microplastic accumulation in the environment and food chain. This has profound consequences such as harm to aquatic life and biomagnification of toxins up the food chain. SUPs are associated with many negative effects on human health such as migration of chemicals into foods and consumption of microplastics. The production and disposal of SUPs also has severe consequences on the environment such as land pollution, water pollution, death of marine animals from ingestion and ecosystem damage. Altogether, these have profound economic impacts such as costs incurred for solid waste management, deterrents to tourism and depletion of limited, non-renewable fossil fuels.

A viable alternative to some SUPs is paper packaging. However, the main challenges associated with paper packaging are sustainability, strength and waterproof capacity. Consequently, full replacement of plastics with paper packaging is not feasible since the functions of packaging are to maintain shelf life and containment. Paper can absorb oil and water but may not be appropriate for long-term applications. There has been dissatisfaction with waterlogging of paper packaging such as paper straws [2]. Consequently, companies have utilized synthetic plastic coatings, which render the packaging not completely biodegradable. Several petroleum-based polymers have been used to coat the surface of paper packaging to increase water resistance, such as epoxies, polypropylene (PP), polyvinyl chloride (PVC) and perfluoroalkyl-based polymers (PFAs) which are not biodegradable. These are toxic and persistent in the environment. Recently, a study showed that paper straws have the highest PFAs as waterproof coatings compared to plastic straws [3].

**Fig 1:**
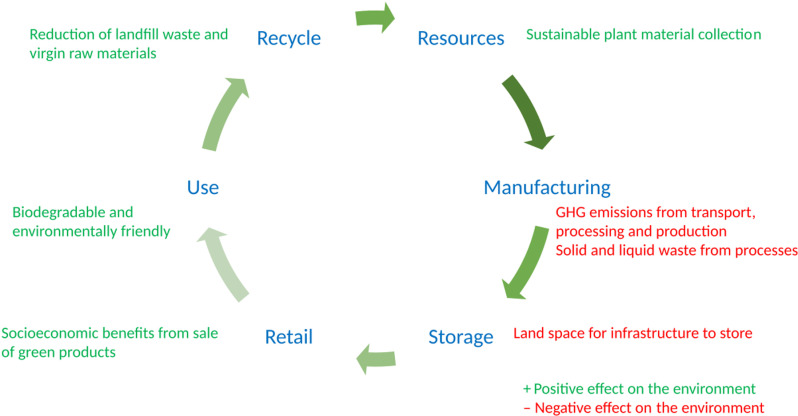
Life cycle assessment for sustainable paper packaging (Adapted from [7]).

Packaging is considered to be sustainable if it has a relatively low environmental impact based on life-cycle assessments (LCA) [2] (Fig 1). While paper has low environmental impacts later on in the life cycle since it is biodegradable, deforestation has a significant impact upstream including its effect on climate change. Many pulp and paper companies are not Forest Stewardship Council (FSC) certified. A cross-cultural consumer study in the United States (US), Germany and France showed that recyclability and biodegradability were prioritized over sustainable raw materials [4]. Sokolova *et al*. [5] reviewed the perceived environmental friendliness of packaging and found that while paper was considered more environmentally friendly, plastic packaging was preferred over plastic-plus-paper-overpackaged counterparts, possibly due to some packaging being considered superfluous. Packaging design and information were also found to be important factors for consumers to recycle [6]

It is important to investigate the Caribbean people’s perceptions of packaging. Conservation psychology requires information about conservation behaviours to positively impact the environment [8]. This can be used by companies and stakeholders involved in making policies to drive sustainable actions [8]. Mission oriented innovation is driven by conceptually sound, theory-driven empirical research [9]. Successful conservation psychology results in conservation marketing which in turn leads to improved social and environmental outcomes, such as reductions in consumption patterns and other unsustainable behaviors [10].

The focus of this paper is on Trinidad as there is a paucity of information on the acceptance of paper packaging in developing countries, including the wider Caribbean. In Trinidad, Peters-Texeira and Badrie [11] performed a baseline study on food packaging, and its impact on food choices using a sample of *n* = 82 from six supermarkets from the perspective of food quality, safety and nutrition facts. Only 4.9% of that sample population found paper packaging to be the most preferred material. There is a need to expand this study to a larger sample size to focus on sustainable paper packaging, especially after new legislation in Trinidad which limits the use of SUPs and styrofoam. The Trinidad and Tobago Bureau of Standards (TTBS) has developed a compulsory standard to support legislation by the GORTT to control the use of expanded polystyrene (EPS) products in the food and beverage sector [12]. This would help to elucidate the receptiveness and awareness of people in Trinidad towards sustainable paper packaging and the understanding environmental impacts of the paper industry.

In the wider Caribbean, Clayton [13] examined the attitudes and practices of teachers, parents and students toward reducing SUP usage in Jamaica. There was a general willingness among the groups to reducing SUP use, recycling, reusing and even banning SUPs. There was awareness that SUPs affected several habitats, including education of children and targeting the different groups were important.

Several studies were done internationally about sustainable packaging in general but some of the more recent studies are highlighted. A study done in Sweden investigated consumer perceptions of packaging functions and material and what is environmentally sustainable packaging and its importance [14]. Material selection for the purpose and recyclability and use of more natural materials were the major factors contributing to sustainability and the majority (75%) agreed that paperboard had the least perceived environmental impact [15]. Another UK study on perception of sustainable food packaging investigated sustainability habits and knowledge, labelling and sustainable packaging attributes [15]. The key findings highlighted the following main quality attributes of sustainable packaging: biodegradability, materials sourced from renewable resources, recyclability, prevention of excess packaging, and product quality. Additionally, labeling plays a crucial role in communicating these attributes to consumers [16].

Oloyede and Lignou [2] investigated sustainable paper-based packaging perceptions in the UK and found that participants were knowledgeable about the types and effects of packaging but the majority were unwilling to pay more for paper packaging since it did not meet expectations. A study done in Germany evaluated the willingness to pay (WTP) for environmentally friendly packaging alternatives finding that unpackaged products had the highest WTP, followed by recycled plastics then paper [16]. Orzan *et al.* [17] conducted a study in Romania and found that persons were aware of the impact of packaging on the environment, and their packaging purchasing decisions were informed by environmental protection, recycling and responsibility with some preference toward paper. There was low WTP also influenced by budget constraints and insufficient information.

A study conducted in Australia found a correlation between packaging concerns among environmentalists, pro-packaging groups, food-waste conscious consumers and companies aiming to minimize packaging [18]. The common factor in all these studies was the tradeoff between multiple packaging attributes [19]. While studies examining either survey data or stress testing exist, few have integrated both approaches into a single study to comprehensively understand reception and marketability within a specific population. This study will assess sustainable paper packaging made from waste foliage, examining consumer and company perspectives to gain a comprehensive understanding of sustainable paper packaging in Trinidad. Market research on the need and demand for sustainable paper packaging in Trinidad comes at a very important time when developing countries are trying to reduce their greenhouse gas (GHG) emissions for their Nationally Determined Contributions (NDCs) with paper packaging being a sustainable alternative to SUPs to reduce the carbon footprint. This research fills the gap in the literature on consumer behaviour and sustainability in developing countries.

### Research questions

The most popular food packaging materials are paper and plastic [20] which are derived from raw materials, produced using largely unsustainable practices. The survey aimed to identify the paper products most preferred by the Trinidadian population, along with the reasons for their acceptance and the barriers hindering the adoption of more sustainable packaging options. This information would guide which paper products would be most suitable for stress testing and areas for improvement of the options currently available. The following broad objectives were broken down into questions based on the gaps in the literature:

1. **Consumer knowledge of paper packaging and the environment:**
  - How is paper packaging used and disposed of?
  - Does the use of paper packaging serve as an alternative to less sustainable packaging?
  - How does the use of paper packaging affect the environment?
2. **Consumer attitudes toward paper packaging and the environment:**
  - What is the importance of environmentally sustainable packaging?
  - How willing are consumers to use paper packaging as an alternative to less sustainable packaging?
  - What strategies do consumers feel are most needed to implement environmentally sustainable packaging?
3. **Consumer practices with paper packaging:**
  - Do consumers have issues when using paper packaging?
  - Do consumers reduce and recycle paper?
  - Do consumers use paper packaging as an alternative to less sustainable packaging?
4. **Stress testing sustainable paper packaging:**
  - To identify customer needs in paper packaging, such as sustainability, durability, or design.
  - To identify any issues or challenges customers face with current paper packaging solutions or competitors.
  - To gather feedback on paper packaging prototypes to refine and improve the design.

The study had two components: primary market research to determine consumer profiles and stress testing of sustainable paper packaging to determine customer discovery in a Trinidadian population.

## Materials and methods

### Ethics statement

Ethics approval was sought from The University of the West Indies, St. Augustine, Trinidad, Campus Research Ethics Committee, (Reference number: CREC-SA.2111/04/2023). A confidentiality statement was provided at the start of the questionnaire to assure participants of the strict privacy of their responses and to adhere to research ethics requirements.

### Questionnaire

A Knowledge, Attitudes, Practices (KAP) questionnaire was developed to gather information from citizens of Trinidad regarding their knowledge and attitudes on paper use in Trinidad. The questions were developed using a mixture of questions motivated by previous studies conducted in developed countries [2,15,16] along with original questions created by the authors. The aim was to gather new data on local knowledge, attitudes and practices, without duplicating existing local research. The survey included a variety of multiple choice and open-ended questions. A pretest questionnaire was validated with 30 participants from various demographic backgrounds and adjusted based on the feedback. Questionnaire-based surveys were disseminated to 300 participants (*n* = 300) in the age range 18 to over 60 years in Trinidad. The KAP survey comprised 38 questions in 4 sections (demographics, knowledge, attitudes and practices). All participants volunteered to complete the questionnaires and were contacted either face to face, via social media or e-mail. No vulnerable or potentially vulnerable participants were used. Electronic responses were collected via a Google Form over a period of six months, and the data were tallied with no information about the participants’ identities being disclosed or traceable.

### Semi-structured interviews

**Fig 2:**
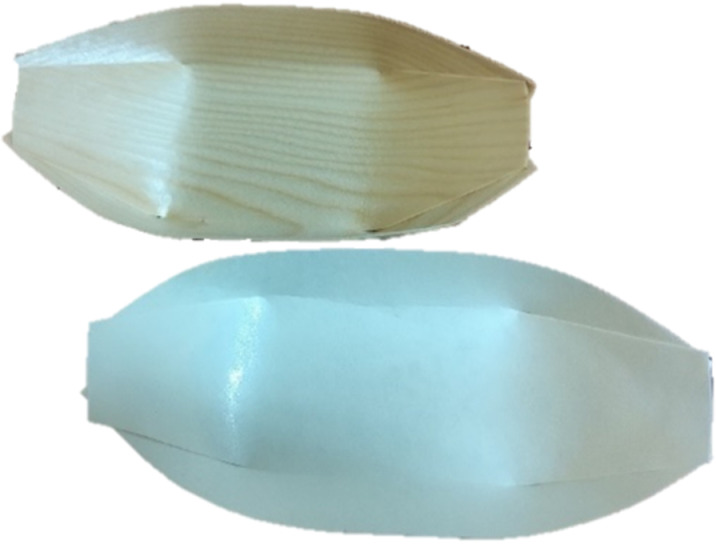
Commercial packaging (above); sustainable non-wood paper packaging (below).

Interviews were also conducted with representatives from packaging companies in Trinidad and Tobago, Jamaica, Barbados, Colombia, Netherlands and Ukraine to understand the reception and challenges to implementing sustainable, waterproof, cutin coated paper packaging made from leaves (Fig 2) using a method protected by trade secret until publication. A mixed methods approach was employed whereby surveys yielded quantitative data for statistical analyses whereas interviews yielded qualitative data from experts. The interviews were not recorded for transcription. Both surveys and semi-structured interviews were used to gather data from consumers and professional opinions from industry persons respectively. Interviews were conducted for 5–15 minutes either face to face or virtually. Local companies were also given the opportunity to view and interact with the sustainable paper packaging.

General questions were asked to elicit pain points and possible solutions.

1. What is the main challenge with packaging?
2. What are some problems with paper packaging?
3. What are some solutions to these problems?
4. What if anything your company has done to address these problems?
5. Are they effective?

### Data analysis

Quantitative data were analyzed using means, standard deviation, and ANOVA. Statistical analyses were done using SPSS (Statistical Package for the Social Sciences) version 29 using descriptive statistics and normality plots for continuous data. The quantitative data were subjected to a one-way ANOVA accompanied by Tukey post hoc test and significant differences among means were established at *p* < 0.05. Regression analysis was done on the KAP scores and correlation of demographics of age, education and environmental consciousness and all the variables that showed significant influence (*p* < 0.05) were selected for multivariate step-wise logistic regression analyses at 95% confidence interval.

Each answer which reflected a positive environmental action was assigned 1 point while incorrect or no responses were assigned 0 points. The knowledge scale ranged from 0 to 11 points, the attitude scale from 0 to 10 points, and the practice scale from 0 to 9 points. The scores were further categorized into low (poor) or high (good) as follows: knowledge (low = 0–5 points; high = 6–11 points); attitude (low = 0–5 points; high = 6–10 points); practice (low = 0–5 points, high = 6–9 points) [21]. Logistic regression analysis was used as well as corresponding 95% confidence intervals.

Qualitative data were tabulated and repeated similar answers were grouped and tallied for trends and represented on bar graphs, pie charts, radar diagram and Krona diagram using thematic and content grouping. A Strengths, Weaknesses, Opportunities and Threats (SWOT) and Political, Economic, Social, Technological, Legal and Environmental (PESTLE) analysis were conducted from the survey responses using grouping of similar answers. The SWOT analysis identifies the advantages and disadvantages while the PESTLE methodology analyzes the political, economic, social, technological, legal, and environmental aspects of technology or innovation from a sustainable perspective [20]. The information from the interviews with industry people was used to create an empathy map. The Empathy Map method is a visual that provides more flexibility than text which is used to describe personas to understand more the end users and for needs assessment [22].

### Packaging testing

The stress test was a separate step after positive feedback in the semi-structured interviews. The need for the stress test was motivated by other international studies [2]. to understand the acceptance in a unique demographic. A semi-quantitative stress test to determine customer perspectives, was also performed in person on the appearance (colour and appearance) and acceptability (practicality and durability) of the paper packaging from leaves (Fig 2) using the Likert scale ranging from very bad (1), bad (2), neutral (3), good (4), very good (5). This was compared to a control brand of biodegradable packaging (*n* = 35). Market analysis was an important step in determining the quality, reliability, and robustness of the sustainable paper packaging compared to a competitor. People from retail outlets in Trinidad who use paper packaging were approached and the two different biodegradable single use packaging presented to the participant. Additional questions from the participants were also answered and participants were allowed to interact with the packaging before making an assessment. The participants who consented were allowed to fill in their responses anonymously using a Google form. This was done over a period of one month, and the data were tallied with no information about the participants’ identities being disclosed or traceable. The average performance ranking was then computed and represented graphically.

## Results

### Demographics

A total of 300 survey responses were collected from Trinidad. Demographic data for the participants (Table 1) showed a fair distribution of gender, education and location (slightly skewed, *p* < 0.05). These factors selected should directly correlate with the knowledge and practices of the participants but there were deviations from the expected (*p* < 0.05). Most of the participants were female (55%, *n* = 164), single (79%, *n* = 237), in the 18–25 year age range (32%, *n* = 95), had a Bachelor’s degree (35%, *n* = 106), from Central Trinidad (27%, *n* = 81) and were environmentally conscious (71%, *n* = 213) (Table 1).

**Table 1:** Demographic Characteristics of Participants (*n* = 300).

Characteristic	Sub-categories	Percentage (%)
**Gender**	Male	45
	Female	55
**Age**	18-25	32
	26-30	23
	31-40	11
	41-50	10
	51-60	14
	>60	10
**Marital Status**	Single	79
	Married	14
	Widowed	1
	Divorced	2
	Prefer not to say	4
**Education**	Primary	0.3
	Secondary	36
	Diploma	12
	Bachelor	35
	Masters	14
	PhD	2.7
**Location in Trinidad**	North	19
	South	20
	East	24
	West	10
	Central	27

### Types of paper packaging and disposal

**Fig 3:**
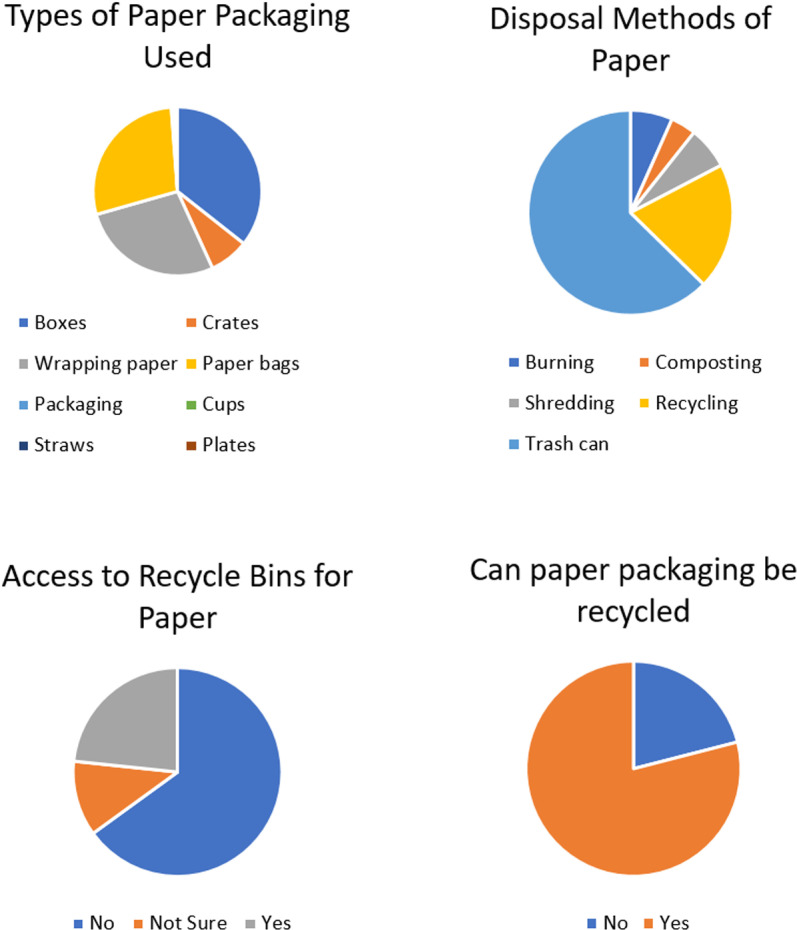
Paper Packaging and Disposal Methods.

Boxes (35%) followed by wrapping paper (27%), paper bags (28%) and crates (45%) were the most popular forms of paper packaging used in Trinidad. The most popular disposal method was in the trash (63%) (Fig 3) followed by recycling (20%) with the minority of participants disposing via composting shredding (7%), burning (7%) and composting (4%). This showed general awareness of the different methods of disposal, but the use of the trash can as the most popular disposal method, can be attributed to convenience. The 20% that recycled paper was far less than the 71% of participants who considered themselves environmentally conscious and 79% that was aware that paper was recyclable. This may be because environmentally conscious persons are more prone to recycled plastic which is not biodegradable and glass which is often seen as a more environmentally friendly packaging material compared to plastics, but its production, transportation impacts, and challenges in recycling can collectively pose significant environmental threats. The 20% of individuals who recycled paper were similarly represented by the 23% of participants who had access to recycling bins specifically for paper.

There were fewer recycling conduits for paper compared to plastic in Trinidad which might explain why 16% of the participants were not sure or aware of the availability of recycled bins for paper and the majority of participants (65%) did not have access to these bins for paper. The access to recycle bins correlated with location which revealed that there was greatest access to recycling bins in the West followed by the East then Central. There is possibly a need for greater distribution of recycled bins in North and South Trinidad. Only 21% of the participants did not think that paper packaging can be recycled. This was not directly correlated with the percentage of people who did not have a tertiary education (*p <* 0.05). This suggested that the perception that paper packaging could not be recycled can be attributed to other factors such as contamination with oils, stains or printing that would render the paper packaging unfit for the recycling process and disposal via trash can, was more suitable.

The distribution of sociodemographic scores for the knowledge, attitude and practices rankings are shown in Tables 2–4 respectively. Linear regression analysis revealed that there was no explained variance between education and knowledge, attitude and practices scores (R^2^ < 0.5). One way ANOVA also (*p* < 0.05) at the 95% CI revealed that there was a significant difference between the knowledge, attitude and practices rankings. Generally, in the Trinidadian population sampled, the knowledge, attitude and practices scores were high (mean > 7) suggesting there is some reception for paper packaging in the population.

**Table 2:** Knowledge scores with sociodemographic characteristics.

Characteristic	Sub-categories	Low	High	*p* value
**Gender**	M	19	117	*p* < 0.12
	F	15	149	
**Age**	18-25	88	7	*p* < 0.28
	26-30	59	9	
	31-40	29	5	
	41-50	26	3	
	51-60	38	5	
	>60	26	5	
**Marital Status**	Single	207	30	*p* < 0.12
	Married	40	3	
	Widowed	7	0	
	Divorced	2	0	
	Prefer not to say	10	1	
**Education**	Primary	0	1	*p* < 0.02
	Secondary	15	92	
	Diploma	4	31	
	Bachelor	8	98	
	Masters	6	37	
	PhD	1	7	
**Location in** **Trinidad**	North	52	6	*p* < 0.003
	South	58	4	
	East	61	9	
	West	21	8	
	Central	74	7	
**Average score**				7.46 (high)

**Table 3:** Attitude scores with sociodemographic characteristics.

Characteristic	Sub-categories	Low	High	*p* value
**Gender**	M	26	107	*p* < 0.06
	F	23	144	
**Age**	18-25	85	10	*p* < 0.01
	26-30	59	9	
	31-40	25	9	
	41-50	20	9	
	51-60	39	4	
	>60	23	8	
**Marital Status**	Single	199	38	*p* < 0.13
	Married	36	7	
	Widowed	6	1	
	Divorced	2	0	
	Prefer not to say	8	3	
**Education**	Primary	1	0	*p* < 0.02
	Secondary	11	96	
	Diploma	2	33	
	Bachelor	31	97	
	Masters	3	40	
	PhD	1	7	
**Location in** **Trinidad**	North	48	10	*p < *0.01
	South	56	6	
	East	59	11	
	West	17	12	
	Central	71	10	
**Average score**				7.08 (high)

**Table 4:** Practice scores with sociodemographic characteristics.

Characteristic	Sub-categories	Low	High	*p* value
**Gender**	M	17	121	*p* < 0.06
	F	6	156	
**Age**	18-25	93	2	*p* < 0.01
	26-30	63	5	
	31-40	32	2	
	41-50	27	2	
	51-60	39	4	
	>60	23	8	
**Marital Status**	Single	217	20	*p* < 0.12
	Married	40	3	
	Widowed	7	0	
	Divorced	2	0	
	Prefer not to say	11	0	
**Education**	Primary	0	1	*p* < 0.06 0.62
	Secondary	100	96	
	Diploma	2	32	
	Bachelor	6	99	
	Masters	0	42	
	PhD	0	8	
**Location in** **Trinidad**	North	52	6	*p* < 0.002
	South	58	4	
	East	67	3	
	West	24	5	
	Central	76	5	
**Average score**				7.29 (high)

### Effects of packaging disposal

**Fig 4:**
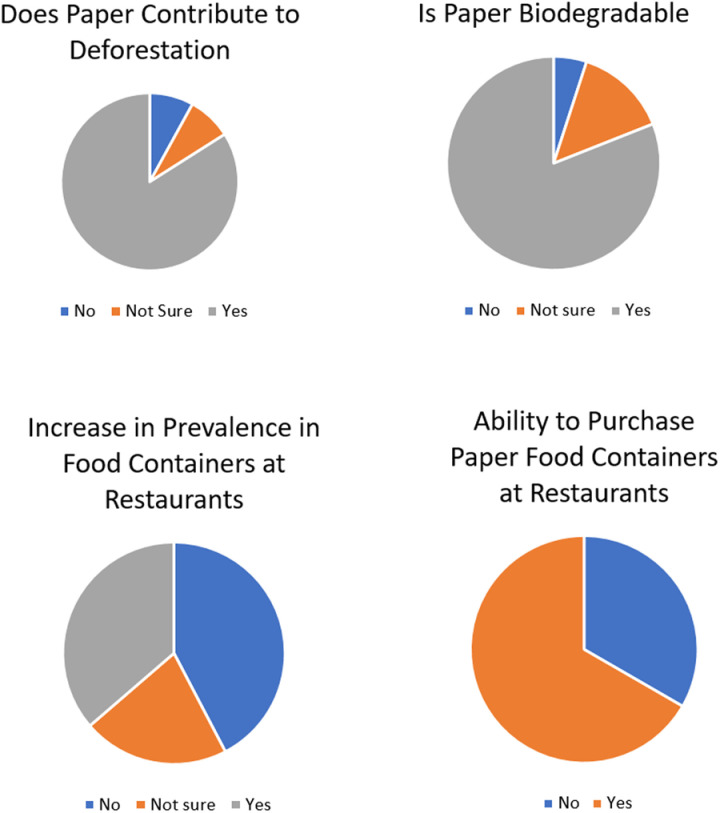
Knowledge about paper contributing to deforestation, biodegradability, prevalence in food industry and purchase.

The majority of participants (84%) correctly responded that paper usage contributes to deforestation as logging is used to obtain the primary raw material (Fig 4). This was not directly correlated with the tertiary educational status (*p* < 0.05). Only 36% of participants found that paper food containers were becoming more popular than Styrofoam containers (Fig 4) following the 2020 ban on importation of Styrofoam [23]. 42% of participants declined, while 21% expressed uncertainty. This suggested a need for increased efforts to replace Styrofoam containers with more durable paper or bioplastic alternatives for takeout.

However, the majority of participants (67%) were willing to purchase paper food containers (Fig 4) which is consistent with 71% of participants who considered themselves environmentally conscious. A regression analysis revealed that education and environmental consciousness together account for less than 50% of the variance in behaviors related to environmental consciousness (R² < 0.5). This indicates a weak to moderate relationship between these factors and the behaviours, suggesting that other influences are likely to affect the outcomes.

### Sources and types of items compatible with paper packaging

**Fig 5:**
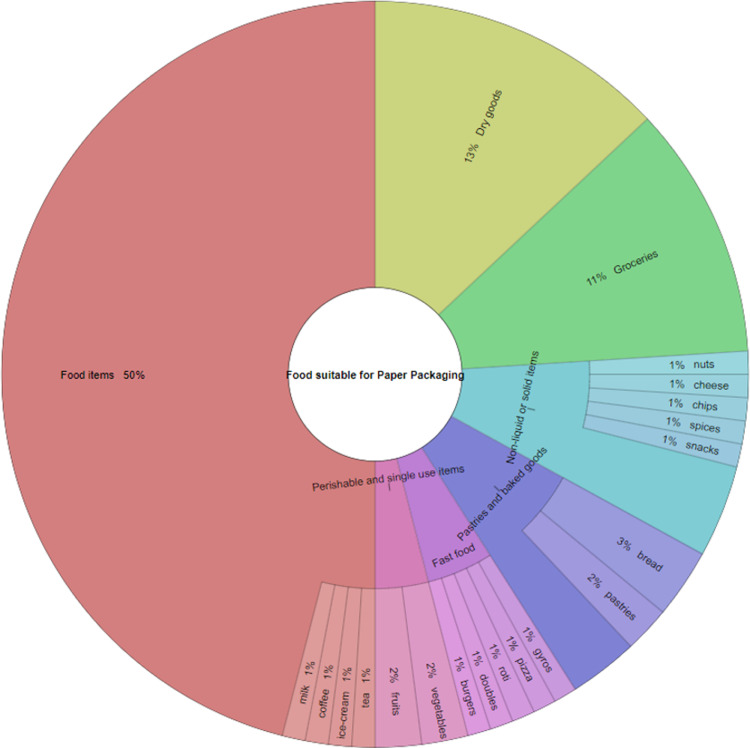
Krona diagram detailing knowledge about the types of foods suitable for paper packaging.

Approximately 49% of the responses encompassed food items as suitable for paper packaging with further descriptors such as non-liquid and dry goods. The Krona diagram (Fig 5) shows the different categories of food in a darker colour, with each broad category being represented by a different colour. In a particular category, the lighter colour shows the resolution of the type of food into quantitative percentages for the categories that were further broken down in the responses. From the Krona diagram, some food items that were deemed as appropriate for paper packaging were pastries, breads, tea, coffee, spices, milk, ice cream, cheese, snacks, chips, nuts, meat, pasta, flour, fruits, vegetables. Fast food items that were already being served in paper packaging were identified such as gyros, doubles, burgers, roti and pizza. Non-food items that were identified as suitable for paper packaging were pads, stationery, jewelry, shipping, clothes and appliances.

**Fig 6:**
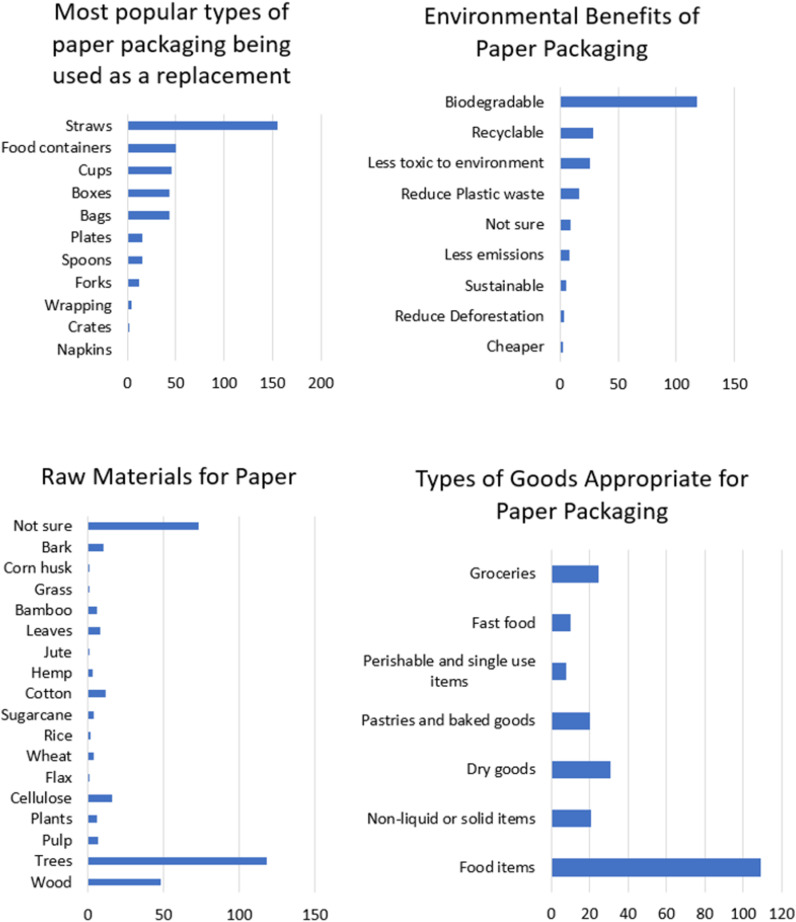
Sources and types of paper packaging. The majority of participants provided a broad description of the source of paper, mentioning trees or plants (39%), while others correctly mentioned logs or specifically cited wood pulp and cellulose (Fig 6). A significant portion of participants were unaware of the source (28%), and some provided incorrect answers like bark. These individuals may not have studied this aspect in science.

Straws (40%), food containers (13%), cups (12%), boxes (11%) and bags (11%) were the most popular identified packaging items being replaced by paper, with less popular identified items being single use plates and cutlery. The first few items serve as good candidates for the sustainable paper packaging, potentially more acceptable to the Trinidadian population. Most of the participants (54%) cited biodegradability as the primary benefit of using paper packaging. Other benefits were that paper was recyclable (13%), less toxic to the environment (12%), reduced plastic waste (8%) and was more sustainable (4%) (Fig 6). Some misconceptions were that paper packaging was cheaper, reduced deforestation and had lower emissions which were contrary to the statistics cited in the literature review.

On the contrary, sugar cane, leaves, grass, jute, cotton, rice, hemp, wheat, flax, corn husk and bamboo were identified as non-wood sources of paper (Fig 6). This might be from more educated participants with higher degrees.

### Attitudes toward the environment and packaging

**Fig 7:**
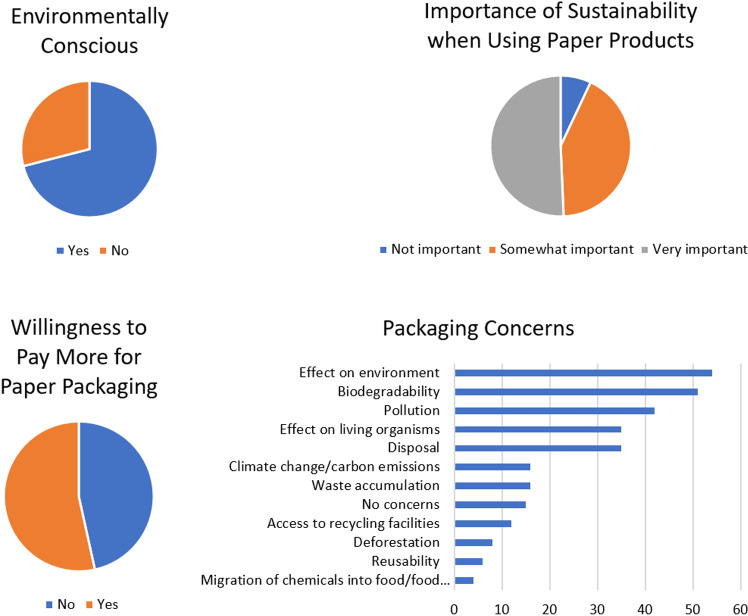
Attitudes toward environmental effects of using paper packaging.

Most of the participants (71%) considered themselves to be environmentally conscious and cited reasons such as awareness, protecting the environment, disliking pollution, participating in beach cleanups, preserving the Earth and recycling habits. The remaining 29% who deemed themselves not to be environmentally conscious indicated they were not doing enough, could not afford it, did not research, did not change habits and did not care. The key packaging concerns were biodegradability (17%) and pollution (32%) (Fig 7). For the importance of sustainability when using paper products most participants agreed that this was either very important (50%) or somewhat important (42%) while the minority (7%) did not find it to be important. These findings were well aligned with the environmental consciousness of the participants. However, there was a notable disparity between the proportion of individuals who were environmentally conscious (71%) and those willing to pay extra for paper packaging (53%).

**Fig 8:**
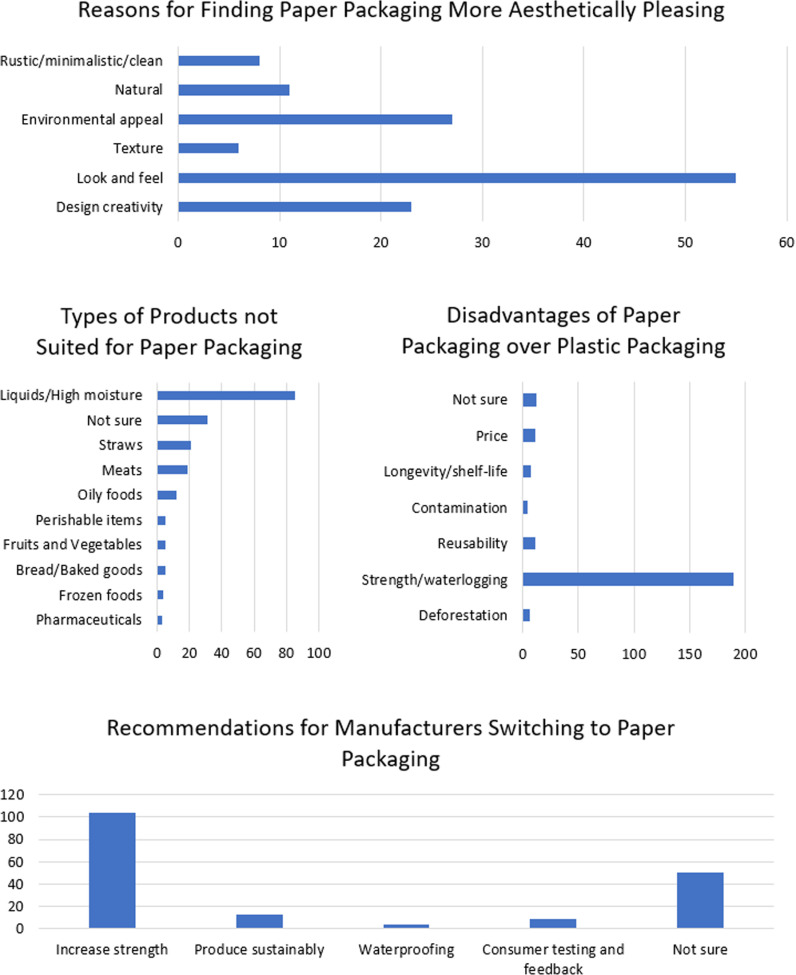
Opinions about the aesthetics and utility of paper packaging compared to plastic packaging.

The aesthetics and quality are important features which influence the acceptance of paper packaging. Most of the participants (73%) indicated that paper packaging was more aesthetically pleasing than plastic which directly correlated with the proportion of participants who considered themselves environmentally friendly (0.64). This was largely attributed to the appearance and texture of the paper packaging (42%) with some individuals elaborating that it appeared rustic, minimalistic, clean, well-designed, had an appealing texture, looked natural and appeared less cheap (Fig 8). The majority of participants (55%) did not notice a difference in the quality of products packaged in paper compared to plastic packaging. The majority of participants (45%) also felt that liquids and high moisture foods were not well suited to paper packaging. This was further validated by persons complaining of strength/waterlogging issues (50%) with paper packaging compared to plastic packaging. Other issues cited were price, longevity, contamination, reusability and deforestation (Fig 8). While the majority of the participants (40%) did not respond or was not sure about the recommendations for manufacturers switching to paper packaging (17%), some important considerations were raised. Most participants (35%) indicated to increase the strength followed by more sustainable production (4%), waterproofing (1%) and consumer testing and feedback (3%) (Fig 8). These issues were taken into consideration and addressed in the current study for the stress test.

### Practices pertaining to the use of paper packaging

**Fig 9:**
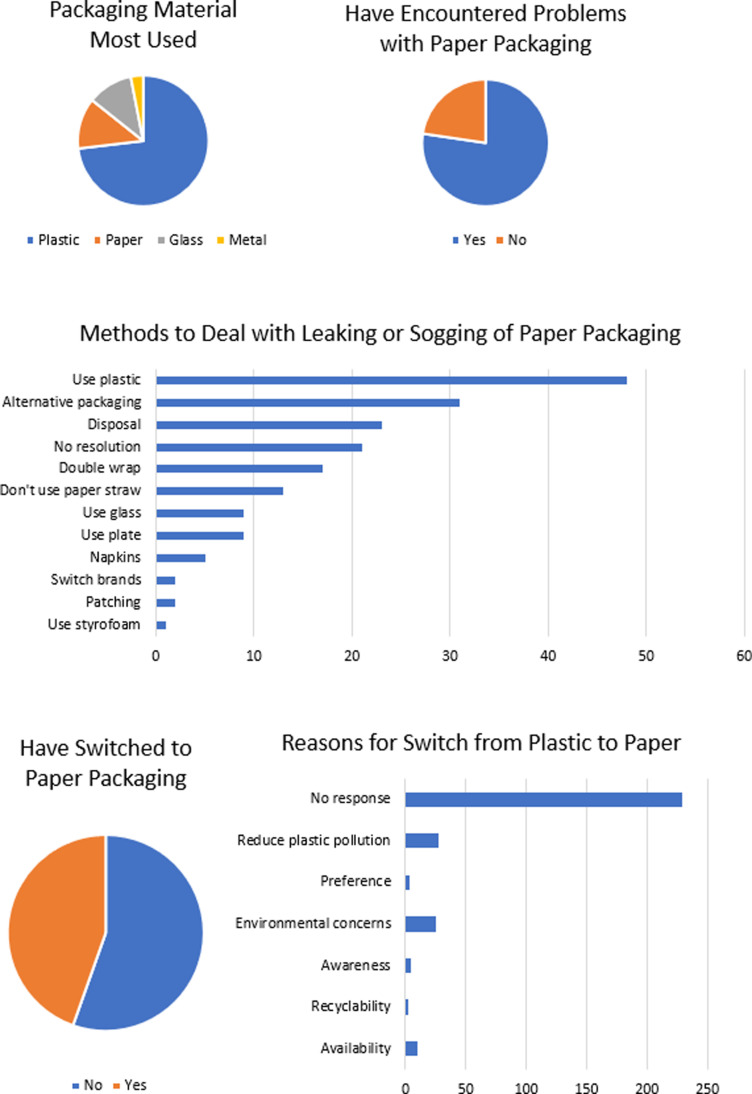
Practices pertaining to the use of paper packaging.

Plastic (72%) was the most widely used packaging material followed by paper (12%), glass (11%) and metal (3%). Most participants (77%) experienced problems with paper packaging and a similar proportion of participants (71%) consequently did not make the switch to paper packaging. For the participants that did make the switch, this was motivated by the need to reduce plastic pollution, environmental concerns, availability, awareness, preference and recyclability. Various methods were employed to address leaking or sogging in paper packaging including the use of plastic (27%), alternative packaging (17%), disposal (13%), no resolution (12%), double wrapping (9%), not using paper straws (7%), using glass (5%), using ceramic plates (5%) and patching (1%) solutions (Fig 9).

**Fig 10:**
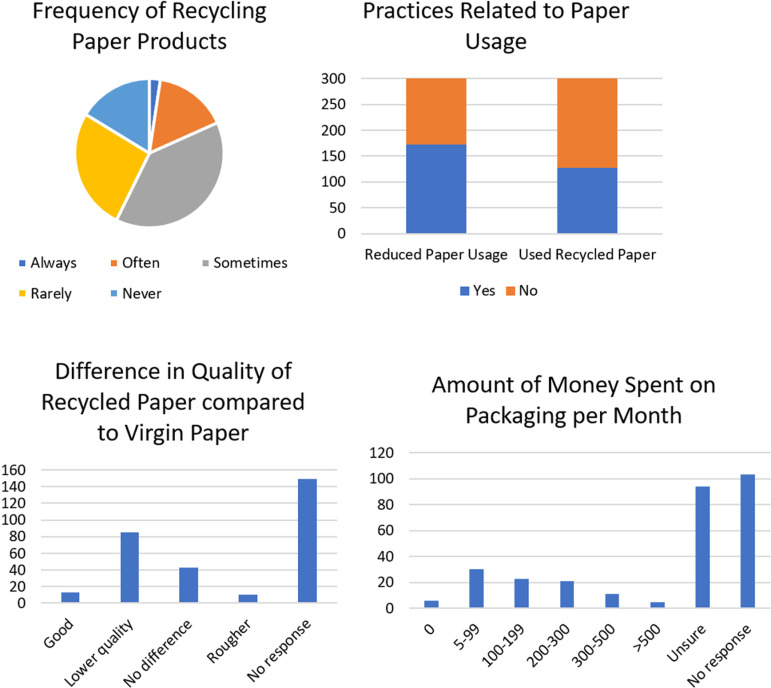
Recycling, use of recycled paper and paper packaging spending.

The majority of participants only recycled paper sometimes (39%) (Fig 10). This may be because the paper is soiled or unrecyclable after food use. Printing paper might be more widely recycled. Interestingly, the proportion of participants who reduced their paper usage (58%) was the same proportion of participants who did not use recycled paper. Although most individuals did not respond to the question about the difference in quality between recycled paper and virgin paper, those who did mention varying perceptions such as lower quality, no difference, rougher texture and good quality (Fig 10). The majority of participants (35%) were unable to estimate the amount of money spent on packaging per month. Answers ranged from $0 TTD to $3,000 TTD, with the majority spending $5–99 TTD on packaging (Fig 10). This suggests that there is a significant demand for packaging in Trinidad, including single use items, which could be targeted for more sustainable alternatives.

### Interviews

The interviewed persons comprised managers from local (6), regional (2) and international packaging (3) companies and engineers (4) corresponding to a total of *n* = 15 professionals.

Identified issues were consistent with customer feedback, such as waterlogging, while non-wood paper packaging companies acknowledged sustainability concerns. Generally, the professionals identified biodegradable, waterproof coatings as a necessary gap in the market to improve the strength of paper packaging. Many companies indicated that they were not currently addressing these challenges. International companies saw the usefulness in a sustainable source of cellulose for paper from non-wood sources as their companies were already doing this. The concept of paper from leaves was considered to be relatively innovative by local professionals. Locally, professionals were impressed by the colour and appearance of the paper packaging. Some persons also asked about the scalability of the methods to different leaves.

### Stress test comparing commercial and sustainable paper packaging

**Fig 11:**
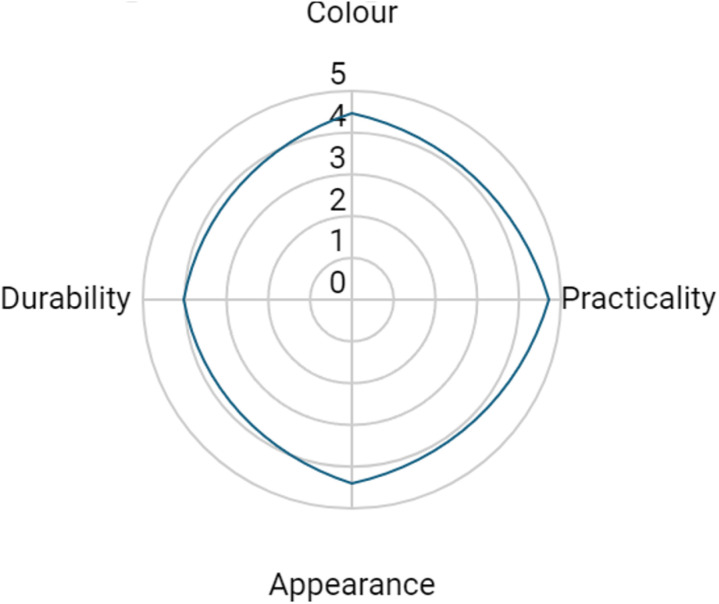
Radar diagram capturing customer perceptions of the biopolymer coated paper packaging from leaves.

Some subjectivity was introduced as the survey was done in the presence of the researcher, despite it being anonymous. There may have been higher scoring due to this (Fig 11). The practicality (4.7) and appearance (4.4) ranked highly. However, the colour ranked slightly lower since there was no real preference over what was considered to be a good colour. Colour did not impact quality and practicality. The lowest ranked attribute was durability. This was because it would not be as strong as the plastic counterpart and in the stress test, a wide variety of foods with different moisture contents, for example, were not shown. Persons also interacted with the material and observed that it was very flexible which may impact the ability to hold heavy items compared to other available packaging. Overall, the stress test showed that there was general acceptance (4.3) of the biopolymer coated paper packaging from leaves. The results could have been improved by using a larger sample size.

**Fig 12:**
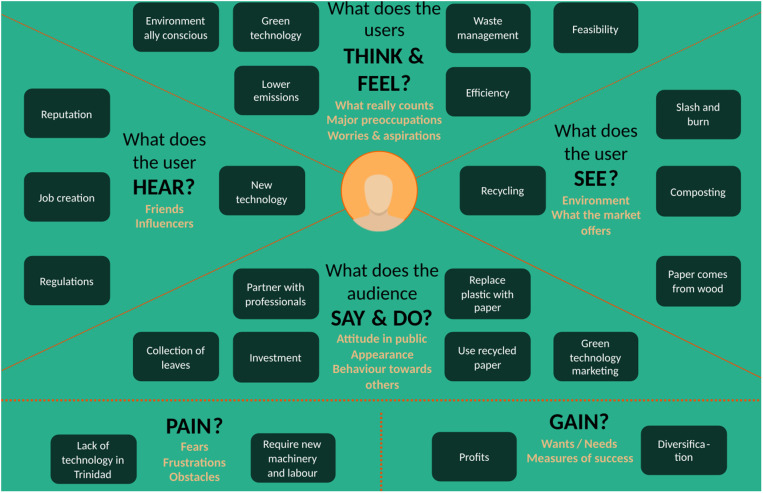
Empathy map for end user of paper packaging.

Based on the results of the survey, focus groups with three paper processing industries, and stress testing, an empathy map and SWOT analysis were constructed to gauge the positioning of the new proposed technology in a Trinidadian market. The empathy map (Fig 12) presents the challenges and gains with the implementation of paper from leaves which presents a new standpoint in the literature.

**Fig 13:**
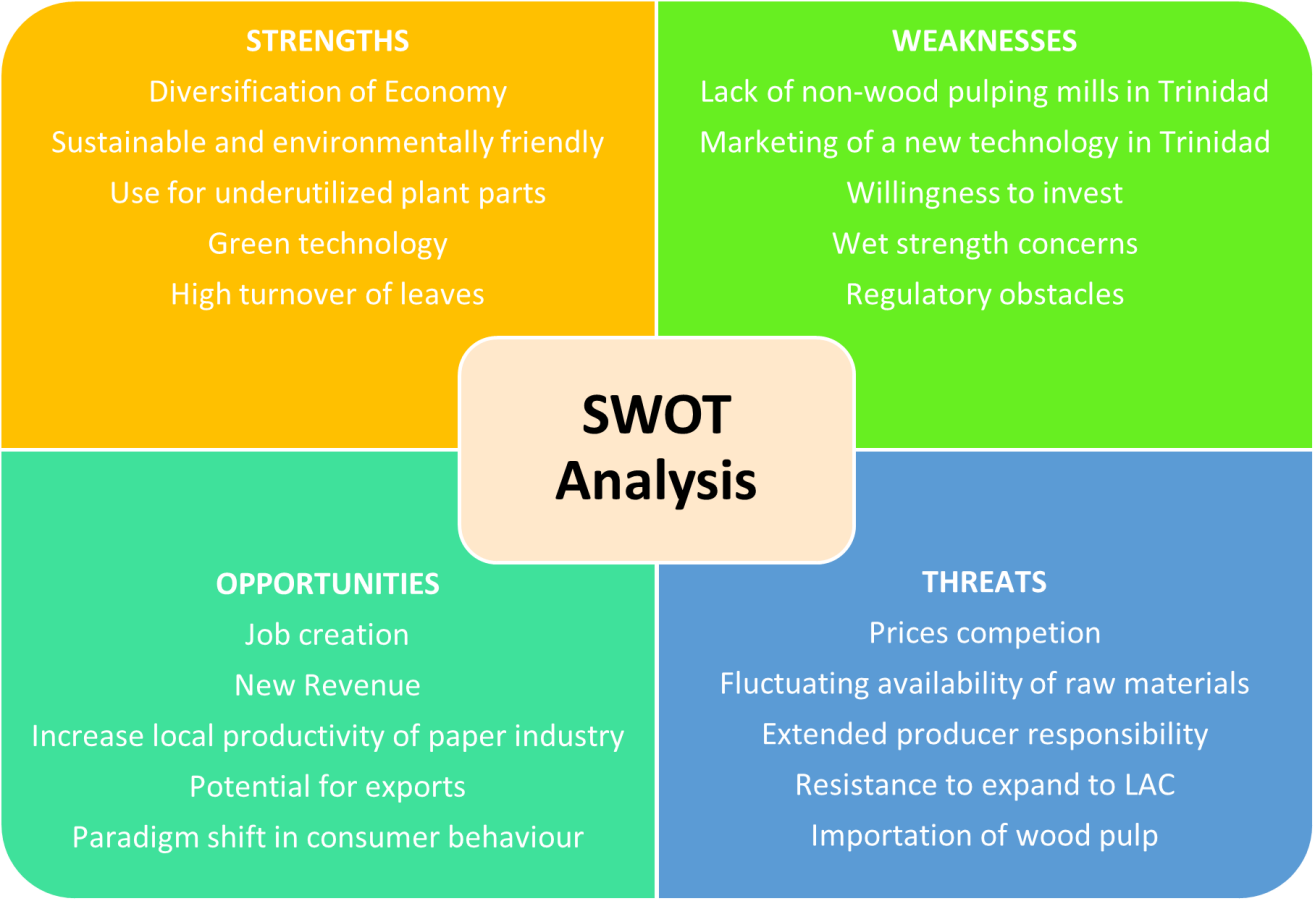
SWOT analysis of paper industry in Trinidad.

SWOT analysis is a framework used to evaluate the strengths, weaknesses, opportunities, and threats of the proposal. PESTLE analysis is a framework used to evaluate the political, economic, social, technological, legal and environmental factors involved. Fig 13 shows the SWOT analysis and Table 5 shows the PESTLE analysis of the paper packaging industry in Trinidad. These analyses offer a structured approach to assess the pros and cons of the switch from SUPs to paper packaging in the unique geopolitical landscape in Trinidad. The significance of these analyses includes a structured framework for strategy development, risk assessment, competitive analysis and resource allocation to overcome weaknesses and threats. The importance of these tools is informed decision-making based on conservation psychology.

**Table 5:** PESTLE Analysis of paper industry in Trinidad.

Political	Economic	Social	Technological	Legal	Environmental
Export and Import policies	Fluctuations in prices	Increased environmental awareness	Advancement in processes and technology	Patents and IP rights Environmental regulations that protect forested areas	Collaborations with farmers for foliage collection Reduce carbon footprint and logging

## Discussion

There is a deficiency in the perceptions by the Trinidadian population towards sustainable paper packaging as a replacement to plastics, against the backdrop of pressures from environmental groups and sustainable development goals. The study aimed to determine this market demand for paper packaging and understand the issues associated with its use in order to create a product to circumvent these concerns.

### Demographic composition

The demographic analysis indicated a relatively balanced distribution of participants in terms of gender, education, and location. However, a slight skewness was observed (*p* < 0.05), suggesting that some groups were overrepresented in the sample. Specifically, females made up 55% of the participants, with the majority of participants being single (79%) and falling within the 18–25 year age range (32%). The highest level of educational attainment among participants was predominantly a Bachelor’s degree, held by the majority of participants (35%). Furthermore, the concentration of participants from Central Trinidad (27%) highlighted a geographic imbalance in the sample.

### Implications of demographics on knowledge, attitudes, and practices

The demographic characteristics of the participants seemed to affect their knowledge, attitudes, and practices (KAP) regarding paper packaging and disposal. The predominance of younger, educated, and environmentally conscious individuals (71%) may account for the overall high KAP scores (mean > 7). However, the observed inconsistencies in behaviors—such as a lower percentage of recycling despite high environmental awareness—underscore the gap between knowledge and actual practices.

The main uses of paper packaging were functional purposes and containment and disposed of mainly by disposal due to soiling and limited access to recycle bins. Based on the results, the use of paper packaging can serve as an alternative to less sustainable packaging in Trinidad. The main effect of paper packaging on the environment was identified by most as unsustainable logging leading to deforestation. However, efforts have been increasing to get Forest Stewardship Council (FSC) certification.

The importance of environmentally sustainable packaging was recognized to reduce pollution and reliance on nonrenewable resources. The participants were willing to use paper packaging as an alternative to less sustainable packaging but generally not at an additional cost incurred. Most participants valued strength (35%) but also waterproof capacity and market research as most needed to implement environmentally sustainable packaging. These customer needs determined in the questionnaire were corroborated with the interviews.

The main issues that consumers had when using paper packaging were strength and waterlogging. A small proportion of the participants reduced (58%) and recycled (39%) paper usage, but possibly more efforts are used to recycle non-biodegradable waste such as plastic bottles. Participants used paper packaging as an alternative to less sustainable packaging. The stress testing on the paper packaging prototypes determined that practicality and appearance were the most significant attributes considered for commercial viability.

It was unexpected that the bivariate linear regression did not explain the variance which may be because a skewed population sample was used, or insufficient variables were considered in the study.

### Comparison with previous research

Trinidad has a general willingness to accept paper packaging as a replacement to some plastic packaging alternatives, similar to other studies. Specific patterns that were common to other countries were recycling, willingness to switch from SUPs to paper packaging, concerns with packaging quality and low environmental impact of paper packaging [2,15]. New insights were gleaned from the interviews with packaging companies and stress testing of the non-wood paper packaging. However, previous studies stress tested their paper packaging with perishable food for a longer timespan rather than single use packaging [2]. The quantitative scoring of the knowledge, attitudes and practices which was done in unrelated studies, was a unique approach to subjectively rate the perceptions of persons towards paper packaging. Additionally, a contribution to the existing body of local knowledge is the conservation psychology applied to understanding the paradigm shift in consumer behaviours [11].

### Implications of the findings

The findings are useful for policy making by encouraging the switch from plastic to paper more mandatory, especially in the food industry. The United Nations Environment Programme (UNEP) circular economy approach involves stakeholders, businesses and consumers. The circular economy supports the three pillars of sustainable development: environmental, economic and social development [24]. Solutions such as this are designed to be more efficient in our local environment and achieve sustainable development goals. There needs to be a shift in the mindset among packaging producers and consumers by moving away from non-biodegradable packaging towards sustainable packaging options throughout the value chain. Repurposing and reducing are target areas in this approach.

Theoretical principles can be applied to sustainable packaging solutions by utilizing local biomass as opposed to nonrenewable sources as suggested in this study. The challenge is to develop new clean technologies that can convert substantial amounts of plant material in a low cost and sustainable way, into commercially viable biomaterials to satisfy the needs of our technological society toward the Advanced Bioeconomy (ABE). This can be accomplished by applying circular engineering and the Zero Waste Technology (ZWT) via the efficient use of resources such as energy and water and reduction of waste at all stages of the process [25]. Sustainable bioeconomy (SBE) and circular bioeconomy (CBE), need to consider the sustainable use of biomass for multiple uses including biopolymers, and their commercial materials. Processing can be integrated to the existing value chains through the development of new products, or novel sustainable processes can create new value chains stimulating new product development (NPD).

At each step in the life cycle assessment (LCA) from cradle to grave (Fig 1), output factors are considered such as energy consumption, greenhouse gas (GHG) emissions, waste and recyclability. Sustainably sourced raw materials reduce these environmental impacts. Education and awareness about the importance of sustainable packaging to the environment can increase the uptake and acceptance in developing countries. The lack of strict legislation in developing countries to increase the willingness of companies to switch to biodegradable packaging hinders cradle to cradle packaging assessments. Since there is a knowledge and understanding of the importance of biodegradable packaging, more options can be made available to consumers to make it easier for them to integrate into their practices.

## Limitations to the study

There were several limitations to the study. Sampling bias may have occurred if the survey sample was not representative of the broader population, which could have skewed the results. Response bias is another concern, as participants might provide socially desirable answers, misunderstand questions, or intentionally mislead, distorting the results. Participants may not always offer accurate information, particularly when questions are sensitive or personal. The depth of the study was limited, making it difficult to capture the full complexity of participants’ thoughts or experiences except in open-ended questions. Low response rates, especially for the later questions, could have led to skewed data or low statistical significance. The overlap of knowledge and attitude questions might have caused ambiguity or confusion, affecting the quality of responses. There was limited flexibility in using a survey instrument rather than an interview, as the fixed set of questions, making it harder to adapt and explore emerging topics or insights not considered in the survey design. Additionally, participants may not have taken enough time to answer thoughtfully, particularly since the survey could be seen to be too long or tedious.

## Recommendations

Future research can build on the current findings, and the questionnaire can be replicated after the introduction of sustainable non-wood paper packaging. Follow-up surveys can be conducted, and statistical techniques (such as weighting responses or imputation) can be used to correct for non-response bias. Focus groups can be incentivized and more detailed focus groups are needed to capture additional data, with a larger, random stratified sample size. Question design can be improved by using clear, neutral wording and employing a wider variety of question types (multiple choice, Likert scales, open-ended) to capture diverse responses. Skip logic can be applied to customize the flow and make the survey easier to complete. The updated survey can also be extended to the wider Caribbean to determine if the data is applicable in other SIDS. Focus groups with larger sample sizes and greater interaction with sustainable packaging can provide more insights and identify areas for improvement.

## Conclusion

Paper packaging has emerged in developed countries as a viable source of biodegradable packaging to reduce the reliance on plastic and its environmental impacts. Key stakeholders including consumers, manufacturers, law and policy makers, and farmers each play an essential role in the value chain to ensure the successful transition towards sustainable paper packaging. Understanding the need for this shift across these groups is a crucial step in motivating both companies and consumers to embrace the change from an economic and environmental standpoint. Our study highlighted a general awareness and acceptance of paper packaging in Trinidad, a developing nation, but also emphasized that greater willingness to act is necessary to facilitate a paradigm shift toward sustainable paper packaging. Customer discovery revealed strong support from consumers, manufacturers, and retailers for paper packaging as an alternative to less sustainable options, along with strategies for implementation that align with the objectives of this study and show promise for new market opportunities. Caribbean companies are increasingly aware of the negative environmental impacts of both the paper and plastic industries. However, action is needed to address these effects, such as adopting sustainable paper packaging and reducing waste. Stricter legislation could accelerate change in small island developing states (SIDS), driving efforts to promote a circular bioeconomy. While other countries have made significant progress in transitioning from plastic to paper, SIDS are well positioned to follow suit, given the widespread potential for sustainable paper packaging. In tropical countries, the abundance of foliage year-round provides an ideal resource to replace single use plastics (SUPs) and reduce plastic waste, which harm the surrounding oceans. SIDS are well-positioned to leverage their bioeconomies, replacing SUPs with sustainable alternatives. However, for this to happen, greater awareness, innovation, and policy action are needed to overcome the barriers to adoption. Ultimately, the combined efforts of all stakeholders can help foster a circular bioeconomy, reducing waste, creating new economic opportunities, and protecting the environment.

## Supporting information

S1 Appendix Raw Data for KAP Survey. (XLSX)

S2 Appendix KAP Scores. (XLSX)
